# Targeted Metagenomics of Retting in Flax: The Beginning of the Quest to Harness the Secret Powers of the Microbiota

**DOI:** 10.3389/fgene.2020.581664

**Published:** 2020-10-27

**Authors:** Christophe Djemiel, Estelle Goulas, Nelly Badalato, Brigitte Chabbert, Simon Hawkins, Sébastien Grec

**Affiliations:** ^1^Univ. Lille, CNRS, UMR 8576 – UGSF – Unité de Glycobiologie Structurale et Fonctionnelle, Lille, France; ^2^Université de Reims Champagne Ardenne, INRAE, UMR FARE A 614, Reims, France

**Keywords:** cell wall, flax, soil, meta-omics, microbiota, holobiont, natural fibers, retting

## Abstract

The mechanical and chemical properties of natural plant fibers are determined by many different factors, both intrinsic and extrinsic to the plant, during growth but also after harvest. A better understanding of how all these factors exert their effect and how they interact is necessary to be able to optimize fiber quality for use in different industries. One important factor is the post-harvest process known as retting, representing the first step in the extraction of bast fibers from the stem of species such as flax and hemp. During this process microorganisms colonize the stem and produce hydrolytic enzymes that target cell wall polymers thereby facilitating the progressive destruction of the stem and fiber bundles. Recent advances in sequencing technology have allowed researchers to implement targeted metagenomics leading to a much better characterization of the microbial communities involved in retting, as well as an improved understanding of microbial dynamics. In this paper we review how our current knowledge of the microbiology of retting has been improved by targeted metagenomics and discuss how related ‘-omics’ approaches might be used to fully characterize the functional capability of the retting microbiome.

## Introduction

Natural fibers from different plant species have long been used by man to make textiles and are now being increasingly exploited as a viable replacement for synthetic fibers in composite materials. From a biological point of view the fibers used in textiles and composites are single cells, much longer than they are wide and characterized by the presence of a thick secondary cell wall. Such structures can be found in the xylem (wood fibers) and/or associated with the phloem in the outer tissues of the stem of non-woody plants (e.g., the bast fibers of flax, hemp, ramie), as well as in the walls of the fruits or the leaves of some species (e.g., cotton, kapok, sisal). In bast fiber species, the individual fiber cells (elementary fibers) are grouped together to form so-called fiber bundles.

The ‘quality’ of the fabricated textiles and natural fiber composites (NFCs) is determined by both the mechanical and chemical properties of the isolated fibers/fiber bundles and the subsequent industrial transformation process (Müssig, 2010; Summerscales et al., 2010; Bourmaud et al., 2018), and researchers have therefore explored the different factors influencing the quality of the finished products.

It is generally accepted that the mechanical and chemical properties of plant fibers result from fiber morphology (e.g., length, diameter), cell wall composition (i.e., what polymers are present and in what quantities) and organization (i.e., where in the cell wall the polymers are located and how they are organized and interact with one another). At the biological level, these aspects are determined by the combined and coordinated spatio-temporal expression of several hundreds of genes, and a major challenge is therefore to identify which ones are involved in this process and to understand their role(s). As a result, genomic and genetic studies aimed at improving fiber plants have so far mainly focused on genome sequencing and the identification of genes/traits associated with various agronomic traits (e.g., plant height, fiber yield, fiber diameter) or cell wall construction (e.g., cellulose/pectin/lignin biosynthesis). Flax was the second fiber plant to have its genome sequenced (Wang et al., 2012) after hemp (van Bakel et al., 2011) and can be accessed on the phytozome public database^1^. The sequences of other fiber plants followed a few years later, most likely as a result of the size and/or complexity of the genome with cotton, jute and ramie being sequenced, respectively in 2015 (Li et al., 2015), 2016 (Yuan et al., 2016), 2017 (Sarkar et al., 2017), and 2018 (Luan et al., 2018). In parallel, a number of whole genome transcriptomics studies by microarrays and more recently RNAseq, together with targeted qRT-PCR and *in situ* hybridization, have enabled the identification of genes likely to be involved in cell wall polymer biosynthesis in flax (Roach and Deyholos, 2007; Fenart et al., 2010; Huis et al., 2012; Chantreau et al., 2015; Zhang and Deyholos, 2016; Gorshkov et al., 2017; le Roy et al., 2017). Despite the identification of numerous candidates, examples of functional validation of cell wall genes in flax, as in other fiber species – are relatively limited despite the fact that this species can be genetically transformed and mutant populations are available (Wróbel-Kwiatkowska et al., 2007; Day et al., 2009; Chantreau et al., 2013, 2014).

**FIGURE 1:**
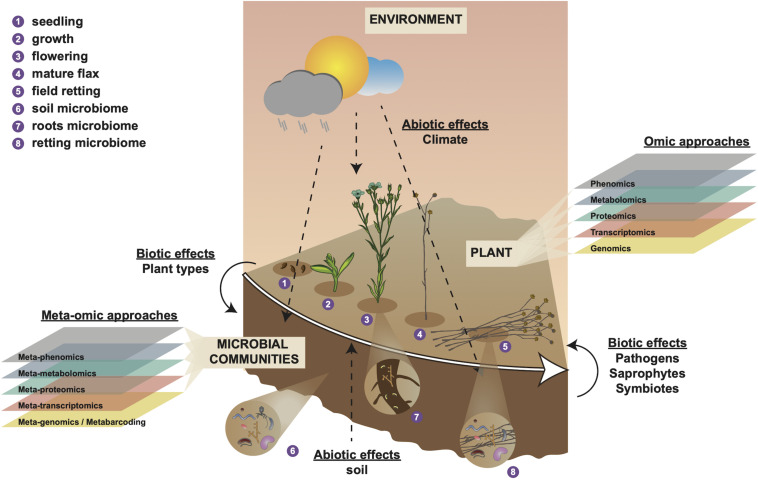
Holistic overview of intrinsic and extrinsic factors contributing to flax fiber phenotype and different scales of (meta-)omic investigation. Numbers 1–4 represent different stages in the development of the flax plant and number 5 represents the retting stage. Number 6 represents the soil microbial community that will interact with plant roots during plant growth (number 7) and with the plant stem during retting (number 8). Biotic effects (**left** side) represent how plant organic material (e.g., leaves) affect soil microbiota. Biotic effects (**right** side) represent how microbiota affect the flax plant during retting (progressive degradation of cell walls). Abiotic effects (soil) represent how soil parameters (e.g., soil type, pH, mineral disponibility, water content) affect the plant during growth. Abiotic effects (climate) represent how meteorological conditions affect plant growth, soil parameters, and microbiome during plant growth (numbers 1–4) and retting (number 5). Omic approaches indicate different scales of investigation that can be used to investigate intrinsic (plant) biology during growth (numbers 1–4). Meta-omic approaches indicate different scales of investigation that can be used to characterize the microbial community during plant growth and retting (numbers 1–5). This diagram underlines the importance of considering the plant-microbiome-environment as a ‘holobiont’ for a complete understanding of how numerous different factors contribute to shaping fiber phenotype.

Linking fiber phenotype (morphology, composition and organization) to gene expression profiles in field-grown flax is an extremely challenging task as these profiles are continually modified during plant growth according to developmental and environmental cues (Figure 1). Both microarrays and RNAseq have been used to investigate modifications in gene expression in response to drought stress (Dash et al., 2014), saline and alkaline stress (Yu et al., 2014), and infection by the fungus *Fusarium oxysporum* (Galindo-González and Deyholos, 2016). In both cases, comparison of the transcriptomes from control plants and stressed plants revealed significant changes in the expression of a number of genes likely to have a direct/indirect impact on fiber phenotype.

The situation is further complicated by the fact that flax fibers are extracted from the stem by mechanical defibering that may alter their mechanical and chemical properties especially at the level of so-called ‘technical fibers’, corresponding to whole or fragmented fiber bundles. Furthermore, mechanical extraction itself is generally preceded by a retting step that can also modify fiber properties if not managed properly (Md.Tahir et al., 2011; Liu et al., 2017).

**FIGURE 2:**
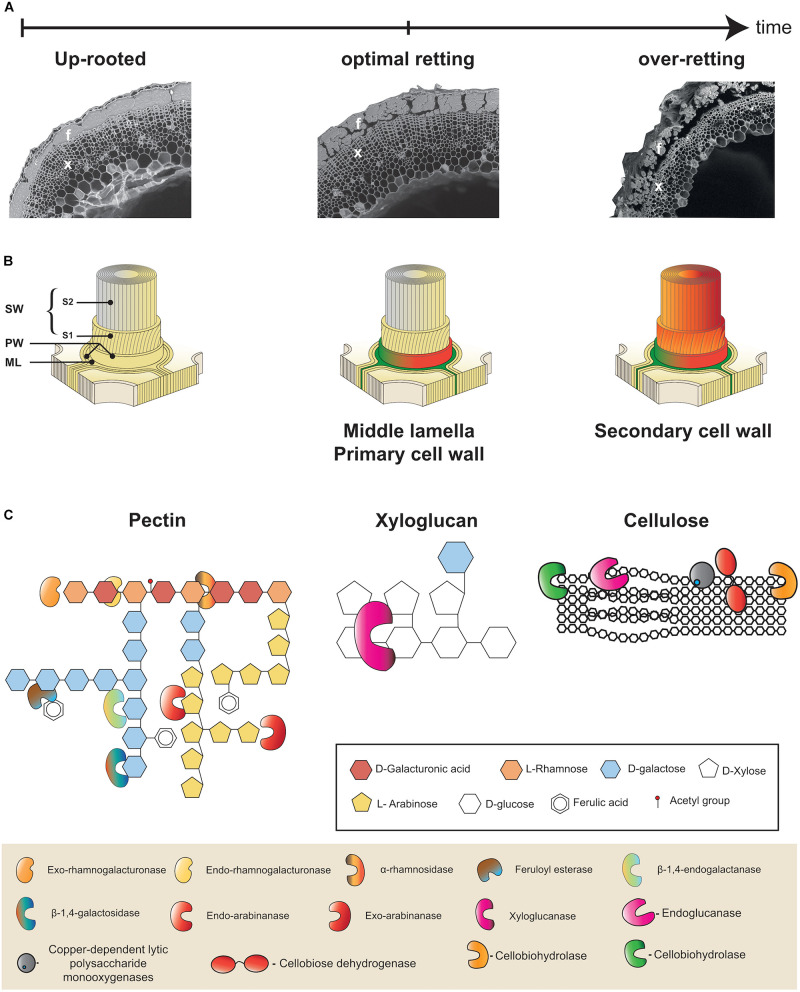
Multiscale presentation of retting process in flax. **(A)** Microphotographs illustrating progressive changes in flax stem morphology during retting. Appearance of a stem cross-section from a freshly up-rooted plant at the beginning of retting **(left)**, at the optimal retting **(middle)**, and over-retted **(right)**. At optimal retting cortical parenchyma cells are no longer visible and fiber bundle dissociation has started. Bast fibers (f) and xylem (x). **(B)** Schematic representation of the different layers of a plant cell wall: middle lamella (ML), primary cell wall (PW), secondary cell wall (SW), S1 and S2 layers of the secondary cell wall (S1, S2). All cell wall layers are intact at the start of retting **(left)**, at optimal retting stage **(middle)** the middle lamella and primary cell walls of cortical parenchyma cells are degraded (green/orange) and the middle lamella between neighboring fiber cells is starting to become degraded (green/orange). At over-retting, the secondary cell wall of fibers starts to become degraded **(right)**. **(C)** Examples of different cell wall polymers: Rhamnogalactan I (RGI) pectin **(left)**, xyloglucan hemicellulose **(middle)**, and cellulose **(right)**. Keys indicate sugar monomers making up polymers (**top** frame) and cell wall degrading enzyme activities (**bottom** frame). This part was adapted from Polizeli et al. (2016).

In field-/dew-retting, plants are pulled (uprooted) and left on the ground for several weeks during which they are colonized by microorganisms (e.g., fungi and bacteria) that produce cell wall degrading enzymes contributing to the progressive de-solidification of fiber bundles from the other stem tissues and partial dissociation of the fiber bundles (Figure 2A). Fibers can also be de-solidified by water-retting, a process in which flax plants are placed into water tanks and colonized mainly by anaerobic bacteria (Tamburini et al., 2003; Zhao et al., 2016). In both cases these natural biological processes affect fiber homogeneity and mechanical performance.

Until recently, our knowledge of the different microorganisms present during retting, the dynamics of colonization, and the complexity of the cell wall degrading enzyme arsenal has been severely limited by our incapacity to cultivate and study the great majority of these species. Furthermore, we know hardly anything about how assembly, activities (i.e., cell-wall degrading capacity) of the retting microbial community and interactions among members are affected by environmental conditions or by plant genotype (Figure 1). The fact that such considerations are important is demonstrated by the results of several studies that have shown that both abiotic factors (e.g., climate, soil properties) and biotic factors (microorganisms) continue to affect the phenotype of plant fibers after harvest during the process of retting (Martin et al., 2013; Liu et al., 2015; Mazian et al., 2018; Bleuze et al., 2020; Chabbert et al., 2020). As a result, retting of fiber species is largely based upon the farmer’s experience and remains a biological black box preventing the development of more objective approaches.

Currently, rapid advances in high-throughput sequencing (HTS) technologies are allowing us to exploit the rapidly expanding field of metagenomics to improve our understanding of retting in flax and other fiber plants (Djemiel et al., 2017; Chabbert et al., 2020). In this paper we review how the application of this approach promises to make a major contribution to the understanding of this complex process and we discuss the perspectives of how related strategies such as metatranscriptomics and metaproteomics could also lead to further advances.

## The Retting of Flax, a Key Step for the Obtention of Quality Fibers

### Retting – Definition and Types

One of the first definitions of retting is as follows: ‘Retting refers to the operation that textile plants are subjected to in order to free the fibers of the liber from the gum-resinous substance which binds them and keeps them attached to the woody stem of the plant’ (Renouard, 1890). The ‘art’ of retting is all about compromise: under-retting will give coarse technical fibers, consisting of several associated individual fibers and contaminated by wood debris and external tissues (cortical parenchyma and cutinized epidermis; Goodman et al., 2002), while over-retting will reduce the resistance of the fibers by affecting their integral structure (Rosemberg and de França, 1967; Akin et al., 1998).

Generally, two main types of retting are used: “water retting” and “field-/dew-retting.” For water retting, flax stems are harvested, grouped in bundles and submerged in water. Historically, this was done in natural basins (e.g., lakes, rivers or dams) for 5–7 days, followed by a period where stems were dried directly on the ground for one to 2 weeks (Akin, 2010). More recently, artificial pits or tanks have been used to wash the stems with clean water to remove residues. This technique makes it possible to control parameters influencing bacterial development including aeration and temperature, and also allows the inoculation of selected bacterial strains. Water-retting depends mainly on the action of anaerobic bacteria that colonize the stem and is related to fermentation (Donaghy et al., 1990). This method generates high quality flax fibers but is also a source of environmental pollution (Md.Tahir et al., 2011). In field-/dew-retting, flax plants are mechanically up-rooted (pulled) by specialized harvesters and the stems placed directly on the field in piles to form swathes (Figure 1; Meijer et al., 1995). Subsequently, the humidity brought by the morning dew and the alternation of rain and heat, favors the development of microorganisms (mainly bacteria and fungi) initially present on the stems and the colonization by the microflora from the soil on which the swathes are placed (Sharma and Faughey, 1999; Akin, 2010; Djemiel et al., 2017). During retting, the intervention of the farmer involves turning the swathes halfway through the process in order to ensure uniform retting over the entire swathe height, and surveying the progression of the process (Franck, 2005; Akin, 2013).

More recently, various alternative methods using commercial enzyme cocktails mainly containing pectinases and xylanases have been investigated in an attempt to provide better control of the process and therefore improve fiber yield and quality (Henriksson et al., 1997; Zhang et al., 2000; Akin et al., 2001). However, their use remains limited mainly because of the high cost of producing these enzymes in bioreactors.

### Retting and Fiber Cell Walls

Individual flax fiber cells (elementary fibers) are grouped together to form fiber bundles that are found in the stem outer tissues surrounding the xylem and pith that constitute the inner tissues (Figure 2A). The bundles are located beneath the epidermis and between the cortical parenchyma and the phloem. During retting, a number of morphological changes occur, mainly in the outer tissues of the stem (Figure 2A). Parenchyma cells around and between fiber bundles that can be easily observed during the early stages of retting almost disappear at later stages (Chabbert et al., 2020). Fiber bundles themselves also become progressively destructured mainly due to the dissociation of individual fiber cells (Akin, 2003; Figure 2A). Individual fiber cells are integrated into stem tissues via their compound middle lamella that not only links them to other fibers, but also links the peripheral bundle cells to the cortical cells in the external stem tissues (Meijer et al., 1995; Akin, 2013; Figure 2B). Retting therefore involves the progressive enzymatic degradation of both cortical parenchyma cell walls and the fiber compound middle lamella. Chemically these structures are composed of different polymers. Cellulose, hemicellulose (mainly xyloglucan), pectic polysaccharides (homogalacturonan, HG), rhamnogalacturonan I (RG-I), and rhamnogalacturonan II (RG-II), as well as some structural proteins are found in the primary cell wall. In contrast, the middle lamella is mainly composed of pectins forming what is commonly called the cellular cement. Fibers cells, in addition to a middle lamella and primary cell wall, have thick secondary walls that are rich in cellulose (up to 80%) and contain non-cellulosic polysaccharides such as galactans and glucomannans (Morvan et al., 2003; Rihouey et al., 2017). Low levels of lignin (2–5%) can also be sometimes found in the middle lamella, primary cell wall, and S1 layer of the secondary cell wall (Day et al., 2005). The technical properties of fibers are due to a high content of crystalline cellulose, with microfibrils almost parallel to the main axis of the cells (Müller et al., 1998). The Figure 2B shows the general organization of the plant cell wall.

### Retting Enzymes

The degradation of cell wall polymers necessitates the intervention of enzymes belonging to a wide range of different enzyme families (Figure 2C). In recent studies on hemp and flax retting, exopolygalacturonase and α-L-arabinosidase, β-D-xylosidase and β-D-galactosidase, β-D-glucosidase and cellobiohydrolase activities were followed during the retting process (Bleuze et al., 2018; Chabbert et al., 2020). Pectin-degrading enzymes are distributed across nine families of carbohydrate-active enzymes (CAZymes), CE8, PL1, PL2, PL3, PL9, PL10, GH28, GH78, and GH88. GH28 polygalacturonases (PGAs) play an important role in the breakdown of pectins in pathogenic ‘fungi.’ Pectin esterases (family CE8) catalyze the de-esterification of pectins into pectate and methanol (Zhao et al., 2013, 2014). Several strains of filamentous ‘fungi,’ isolated from flax stems retted in the field, such as *Rhizomucor pusillus* (Mucoromycota formerly Zygomycota) and *Fusarium lateritium* (Ascomycota) have shown a capacity for decohesion of fibers, in particular thanks to a high level of pectinolytic activity (Henriksson et al., 1997). Pectinolytic activities (e.g., polygalacturonases or pectin lyases) have also been observed in various microorganisms during water-retting (Zhao et al., 2016). For example, *Clostridium felsineum* shows a strong pectinolytic activity and a good capacity for retting. Studies using enzymatic retting have shown that the use of polygalacturonases alone is sufficient for the decohesion of fibers (Evans et al., 2002; Akin et al., 2004). Some microorganisms used during retting such as *Bacillus subtilis* and *Erwinia carotovora* (syn. *Pectobacterium carotovorum*) are known to produce pectin lyases (Sharma, 1986). Saprophytic or parasitic microorganisms have CAZymes showing activities of hemicellulolytic types (vanden Wymelenberg et al., 2010). These activities, in particular those of xylanases, have been observed during water-retting (Donaghy et al., 1990), or in bacterial cultures resulting from the cultivation of dew retted flax stems (Sharma, 1986). Very recently, a study on retting with water in a fermenter, showed a cyclic evolution of mannanases, another hemicellulolytic enzyme (Zhao et al., 2016).

During retting the enzymatic degradation of cell wall polymers can be followed by monitoring the sugars released. For example, the release of fibers was correlated with a decrease in the concentration of galacturonic acid resulting from the breakdown of pectic material in the middle lamella at the end of retting (Rosemberg and de França, 1967). A recent study of retting dynamics by both scanning electron microscope (SEM) imaging and the analysis of certain wall polymers also confirmed the link between the degradation of primary cell walls and fiber middle lamella and the dissociation of fiber bundles (Chabbert et al., 2020). In contrast glucose and mannose/galactose levels remain stable suggesting that secondary cell wall polysaccharides are not degraded (Chabbert et al., 2020). The release of other cell wall polymers such as cutin, wax, and aromatic compounds (sinapyl alcohol and ferulic acid) has also been investigated by chemical analyzes and mass spectrometry in a comparative study of water- and field-retting (Morrison et al., 2000).

### Factors Affecting Retting

At the plant level, the amount of pectins varies depending on the cultivar and will influence the duration of retting (Brown et al., 1986; Haag et al., 2017). Increasing maturity of the stems will also favor lignin deposition in the fiber middle lamella associated with difficulties in retting (Meijer et al., 1995; Pallesen, 1996). However, the influence of exogenous factors, either abiotic or biotic, also has an important impact on retting. In the first category, can be classified all the pedoclimatic factors such as the soil structure or the mineral elements (for example nitrogen, phosphorus and potassium), the physico-chemistry of the soil, the rotation of the cultures, the thickness of the swathe, as well as climatic conditions and seasonal variations (Sharma and Faughey, 1999). In the second category, the most influential factor will undoubtedly be the microbiota present in the soil and in/on the stem at the beginning of retting (Djemiel et al., 2017; Chabbert et al., 2020). Intriguingly, a recent study has revealed the existence of a very close link between the microbiome and the spatial and temporal development of the plant (composition, physiology), or even cultural conditions (Comeau et al., 2020). In the light of such observations it seems likely that plant growth and fiber development in flax will also be affected by the microbiome present. Furthermore, the microbiome itself will be influenced by plant growth and retting conditions. A more integrated approach is clearly necessary if we are to fully understand all of the factors affecting fiber quality (Figure 1).

## Microbiology of Retting, What Have We Learned From Classical Approaches?

Since it is the microorganisms that produce the hydrolytic enzymes responsible for retting, an important step in our understanding of this process is to identify the organisms responsible. Historically, various bacteria and fungi were identified in a number of different studies using isolation and culture-based approaches. However, such strategies are not powerful enough to obtain a complete inventory of the microorganisms present as only a small percentage of taxa can be successfully cultured under laboratory conditions. Moreover, these approaches are generally inappropriate for dynamic studies looking at how microbial communities evolve during retting. More recently, microbial retting studies have greatly benefited from the use of HTS technologies that have produced exhaustive inventories of bacteria and fungi linked with this process. A list of microorganisms identified by classical methods is given in Tables 1, 2 and those identified by metabarcoding approaches in Table 3.

**TABLE 1:** List of different bacterial species involved in flax retting and identified by classical approaches.

**Retting Mode**		**Phylum**	**Current Name (Name in Publication)**	**Localization**	**Method**	**References**
**Water**	**Dew**					
X		Firmicutes	*Bacillus amylobacter*	Not mentioned probably France	Not mentioned	van Tieghem (1879)
X		Proteobacteria	*Enterobacter aerogenes (Bacterium aerogenes)*	Not mentioned	Screening on agar media or liquid culture	Allen (1946b)
X		Proteobacteria	*Escherichia coli (Bacterium coli)*	Not mentioned		
X		Proteobacteria	*Streptococcus* (Streptococci)	Not mentioned		
X		Firmicutes	*Lactobacillus* (Lactobacilli)	Not mentioned		
X		Firmicutes	*Clostridium tertium*	Not mentioned	Screening on agar media or liquid culture	Allen (1946a)
X		Firmicutes	*Clostridium*	Australia (Melbourne)	Not mentioned	Lanigan (1950)
X		Firmicutes	*Clostridium felsineum*	Australia (Melbourne)		
X		Proteobacteria	*Achromobacter parvulus*	Brazil (Santa Catarina)	Screening on agar media culture	Rosemberg (1965)
X		Proteobacteria	*Aerobacter cloacae*	Brazil (Santa Catarina)		
X		Proteobacteria	*Aerobacter aerogenes*	Brazil (Santa Catarina)		
X		Firmicutes	*Bacillus brevis*	Brazil (Santa Catarina)		
X		Firmicutes	*Bacillus cereus*	Brazil (Santa Catarina)		
X		Firmicutes	*Bacillus megaterium*	Brazil (Santa Catarina)		
X		Firmicutes	*Bacillus sphaericus*	Brazil (Santa Catarina)		
X		Firmicutes	*Bacillus subtilis*	Brazil (Santa Catarina)		
X		Firmicutes	*Clostridium butylicum*	Brazil (Santa Catarina)		
X		Firmicutes	*Clostridium beijerinckii*	Brazil (Santa Catarina)		
X		Firmicutes	*Clostridium saprogenes*	Brazil (Santa Catarina)		
X		Firmicutes	*Clostridium sartagoformum*	Brazil (Santa Catarina)		
X		Firmicutes	*Clostridium saccharoacetoperbutylicum*	Brazil (Santa Catarina)		
X		Firmicutes	*Clostridium perenne*	Brazil (Santa Catarina)		
X		Proteobacteria	*Escherichia coli*	Brazil (Santa Catarina)		
X		Actinobacteria	*Gaffkya tetragena* (probably contamination)	Brazil (Santa Catarina)		
X		Proteobacteria	*Pseudomonas aeruginosa*	Brazil (Santa Catarina)		
X		Proteobacteria	*Pseudomonas pseudomallei*	Brazil (Santa Catarina)		
X		Proteobacteria	*Paracolobactrum aerogenoides*	Brazil (Santa Catarina)		
X		Proteobacteria	*Serratia plymuthica*	Brazil (Santa Catarina)		
X		Firmicutes	*Staphylococcus epidermis* (probably contamination)	Brazil (Santa Catarina)		
	X	Firmicutes	*Bacillus mycoides*	Northern Ireland (Lambeg)	Screening on agar media culture	Sharma (1986)
	X	Firmicutes	*Bacillus subtilis*	Northern Ireland (Lambeg)		
	X	Proteobacteria	*Erwinia carotovora*	Northern Ireland (Lambeg)		
	X	Proteobacteria	*Pseudomonas fluorescens*	Northern Ireland (Lambeg)		
	X	Proteobacteria	*Pseudomonas putida*	Northern Ireland (Lambeg)		
	X	Actinobacteria	*Micrococcus* sp.	Northern Ireland (Lambeg)		
X		Firmicutes	*Bacillus subtilis*	Northern Ireland (Lambeg)	Screening on agar media culture	Donaghy et al. (1990)
X		Actinobacteria	*Cellulomonas* spp.	Northern Ireland (Lambeg)		
X		Firmicutes	*Clostridium felsineum*	Northern Ireland (Lambeg)		
X		Firmicutes	*Bacillus cereus*	Northern Ireland (Lambeg)		
X		Firmicutes	*Clostridium felsineum*	Italy	Screening on agar media or liquid culture and identification with 16S rDNA	Tamburini et al. (2003)
X		Firmicutes	*Anaerobacter polyendosporus*	Italy		
X		Firmicutes	*Clostridium saccharobutylicum*	Italy		
X		Firmicutes	*Clostridium aurantibutyricum*	Italy		
X		Firmicutes	*Clostridium acetobutylicum*	Italy		
X		Firmicutes	*Bacillus subtilis*	Italy		
X		Firmicutes	*Bacillus pumilus*	Italy		
X		Firmicutes	*Paenibacillus amylolyticus*	Italy		

**TABLE 2:** List of different fungal species involved in flax retting and identified by classical approaches.

**Retting Mode**			**Phylum**	**Current Name (Name in Publication)**	**Localization**	**Method**	**References**
**Water**	**Dew**	Standing*					
	X		Ascomycota	*Epicoccum nigrum*	Ireland (Lambeg, Hillsborough)	Screening on agar media culture	Brown (1984)
	X		Mucoromycota (Zygomycota)	*Rhizopus* sp.			
	X		Mucoromycota (Zygomycota)	*Mucor* sp.			
	X		Ascomycota	*Cladosporium herbarum*			
	X		Ascomycota	*Botrytis cinerea*			
	X		Ascomycota	*Penicillium* sp.			
	X		Ascomycota	*Fusarium culmorum*			
	X		Ascomycota	*Phoma* sp.			
	X		Ascomycota	*Alternaria* spp.			
	X		Ascomycota	Yeasts			
		X	Ascomycota	*Cladosporium herbarum*	Northern Ireland (Lambeg)	Screening on agar media culture	Sharma (1986)
		X	Ascomycota	*Fusarium culmorum*			
		X	Ascomycota	*Botrytis cinerea*			
		X	Ascomycota	*Epicoccum nigrum*			
		X	Ascomycota	Yeast			
		X	Ascomycota	*Alternaria* spp.			
	X		Ascomycota	*Aspergillus flavus*	Italy (Budrio)	Not mentioned	Fila et al. (2001)
	X		Ascomycota	*Aspergillus niger*			
	X		Ascomycota	*Epicoccum nigrum*			
	X		Ascomycota	*Fusarium oxysporum*			
	X		Mucoromycota (Zygomycota)	*Mucor hiemalis*			
	X		Ascomycota	*Penicillium simplicissimum*			
	X		Mucoromycota (Zygomycota)	*Rhizopus stolonifer*			
	X		Ascomycota	*Fusarium equiseti*	United States (South Carolina)	Screening on agar media culture	Henriksson et al. (1997)
	X		Ascomycota	Yeast			
	X		Mucoromycota (Zygomycota)	*Rhizomucor pusillus*			
	X		Ascomycota	*Trichoderma virens*			
	X		Ascomycota	*Alternaria alternata*			
	X		Ascomycota	*Fusarium lateritium*	United States (Connecticut)		
	X		Ascomycota	*Cladosporium herbarum*			
	X		Ascomycota	*Fusarium oxysporum*	France		
	X		Ascomycota	*Epicoccum nigrum*	Holland		

*Flax plants are retted in upright ‘wigwam’ like piles.

**TABLE 3:** Non-exhaustive list of studies on the microbiology of retting performed using a metabarcoding approach.

**Plant Fiber**	**Retting Types**	**Microbes Studied**	**Molecular Marker Used**	**Sequencer**	**Years**	**OTU Richness (mean)**	**Subsampling**	**Major Phyla**	**References**
Kenaf (*Hibiscus cannabinus*)	Water	Bacteria	16S rRNA	Ion torrent PGM	2013	1,500	28,000	Firmicutes, Proteobacteria, Bacteroidetes	Visi et al. (2013)
Flax (*Linum usitatissimum*)	Water	Bacteria	16S rRNA	Illumina MiSeq	2016	70	NM	Bacteroidetes, Firmicutes, Proteobacteria	Zhao et al. (2016)
Flax (*Linum usitatissimum*)	Dew	Bacteria; Fungi	16S rRNA; ITS2	Illumina MiSeq	2017	300; 220	20,548; 42,436	Proteobacteria, Bacteroidetes, Actinobacteria, Firmicutes; Ascomycota, Basidiomycota	Djemiel et al. (2017)
Flax (*Linum usitatissimum*)	Dew	Bacteria; Fungi	16S rRNA; ITS2	Illumina MiSeq	2020	200; 260	5,919; 86,050	Proteobacteria, Actinobacteria, Bacteroidetes; Ascomycota, Basidiomycota	Chabbert et al. (2020)
Hemp (*Cannabis sativa*)	Greenhouse	Bacteria	16S rRNA	Illumina MiSeq	2020	100	2,234	Proteobacteria, Bacteroidetes	Law et al. (2020)
Kenaf (*Hibiscus cannabinus*)	Water	Bacteria; Fungi	16S rRNA; 18S rRNA	Illumina MiSeq	2020	430*	NM	Bacteroidetes, Proteobacteria; Basidiomycota, Ascomycota	Duan et al. (2020)

*chao1 metric; NM: not mentioned.

### Bacteria Identified

**FIGURE 3:**
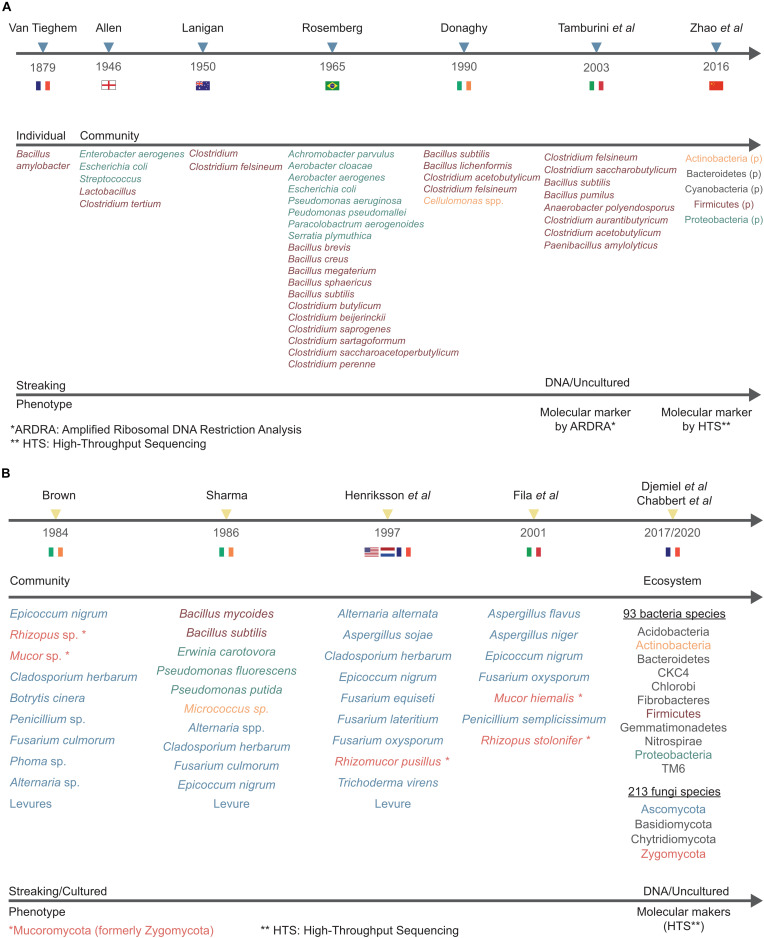
Timelines showing the increasing number of bacterial and fungal species identified over time in flax water-retting **(A)** and dew-retting **(B)**. Figure shows authors and year of published studies (**top** line), microbial organization level (individual, community, ecosystem) and name (**middle** line), culture method and type of analysis (**bottom** line). The color of phyla (HTS approach) and species names indicates the identification method type (classical *vs*. HTS). When phyla names are in black it means that they have been identified for the first time by metabarcoding approaches.

The first reported study focusing on the retting of flax with water was carried out by the French biologist and botanist Philippe Édouard Léon Van Tieghem (1839–1914), who concluded that the bacteria *Bacillus amylobacter* was probably responsible for the decomposition of pectins (Figure 3A; van Tieghem, 1879). The second genus to be associated with water-retting was the genus *Clostridium* and more particularly *Clostridium felsineum* (Figure 3A; Lanigan, 1950). A few years later, Rosemberg (1965) isolated 22 individuals including species of the *Clostridium* and Pseudomonas genera. *Pseudomonas aeruginosa* was recognized as the fastest species in fiber decohesion (Rosemberg, 1965). This study also identified, for the first time, *Achromobacter parvulus* which appears to be involved in the latter stages of retting.

Later, Sharma (1986) isolated and identified six bacterial species from the Actinobacteria, Firmicutes, and Proteobacteria phyla as well as five fungal species belonging to the Ascomycota phylum (Figure 3B).

In 1990, a study at the University of Ulster (Northern Ireland) followed the evolution of anaerobic bacteria during water-retting, both at an industrial and laboratory scale. *Bacillus licheniformis* and *Bacillus subtilis* were the most dominant during the first and second phase, corresponding to the growth phase and the rapid pectinolytic phase (between 10–40 h) of water-retting. *Clostridium acetobutylicurn* and *Clostridium felsineum* appear during the last phase known as the slow pectinolytic phase (Figure 3A; Donaghy et al., 1990). All the bacteria considered belong to the Firmicutes phylum.

In 2003, another study provided further information about pectinolytic bacteria involved in water-retting. Although this study still involved a culture step to isolate bacteria, it was the first one to use a molecular marker approach, in this case by amplifying a partial region of the 16S rRNA gene, using the (amplified ribosomal DNA restriction analysis) ARDRA technique. All of the anaerobic strains were assigned to the *Clostridium* genus and the aerobic strains to the *Bacillus* or *Paenibacillus* genera. Anaerobic colonies with significant polygalacturonase activity belonged to two phylogenetic clusters assigned to the *Clostridium acetobutylicum*/*Clostridium felsineum* and *Clostridium saccharobutylicum* species. For aerobic bacteria, colonies with significant polygalacturonase activity belonged to two phylogenetic clusters assigned to the *Bacillus subtilis* species (Figure 3A; Tamburini et al., 2003). All these strains also belong to the Firmicutes phylum.

### Fungi Identified

One of the first studies on the microbiology of dew-retting identified several genera and species from the Fungi kingdom belonging to the Ascomycota and Mucoromycota (formerly Zygomycota) phyla (Figure 3B; Brown, 1984). In the late 1990s, an American study looked at fungi involved in flax dew-retting in the United States, Netherlands, and France (Figure 3B; Henriksson et al., 1999). Seven strains of filamentous fungi (including six still not described in the literature to this day) and a yeast, could be identified following isolation, cultivation on synthetic media, and purification in order to compare their activities and their efficiency in fiber release. Another study involving *in vitro* retting tests was carried out by Fila and coworkers but no new fungal genus was identified (Figure 3B; Fila et al., 2001).

Some fungi have been characterized as over-retting actors, for example *Fusarium lateritium* and more particularly *Epicoccum nigrum*, recognized as being a primary saprophyte in retting (Brown, 1984; Henriksson et al., 1997; Akin et al., 1998). Another species, *Rhizomucor pusillus* has been observed to degrade part of the surface of the cuticle, thus probably allowing the entry of microorganisms (Henriksson et al., 1997).

The overall conclusion that can be drawn from these classical, culture-based studies of retting is that the type of microorganism identified depends heavily upon the retting type. Field retting is an aerobic environment and although both bacteria and fungi are identified it is generally the latter that predominate with species belonging mostly to the Ascomycota phylum and to a lesser extent the Mucoromycota phylum (formerly Zygomycota; Table 2). In contrast, water-retting mainly involves anaerobic microorganisms (bacteria). Nevertheless, several fungi have also been identified in various anaerobic environments and classified within two very close phyla, Neocallimastigomycota and Chytridiomycota (Griffith et al., 2010; Gruninger et al., 2014). Interestingly, some of these anaerobic fungi, are also found in the rumen, and have been shown to possess a number of GH genes probably resulting from horizontal transfers of bacterial genes (Garcia-Vallvé et al., 2000; Steenbakkers et al., 2001).

## Metabarcoding and the Breakthrough in Knowledge on Retting Microbial Diversity

**FIGURE 4:**
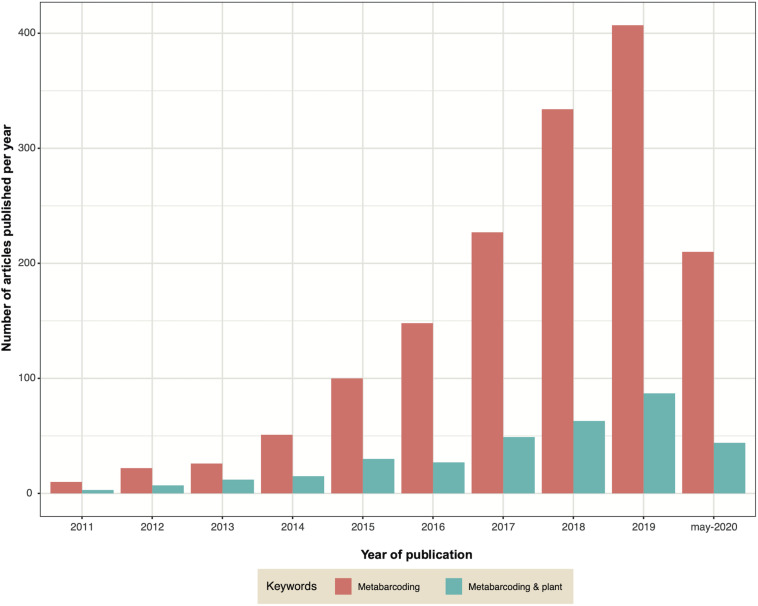
Graph indicating the number of publications containing the keywords ‘metabarcoding’ (pink) or ‘metabarcoding AND plant’ (green) published each year since 2011.

Metabarcoding, also known as targeted metagenomics, is an approach that has revolutionized the study of microbial communities. In recent years the decrease in sequencing costs has allowed it to become more democratic, leading to the use of this approach in a larger number of laboratories and studies. The large and growing number of publications that use metabarcoding reflects this evolution regardless of the habitat explored, including studies on the microbiota of plants (Figure 4). Metabarcoding consists in sequencing an amplicon (most often corresponding to one or more regions of a phylogenetic marker) on a high−throughput platform to characterize microbial diversity from complex/environmental samples, thereby enabling studies of alpha-diversity, community structure or taxonomy (Deiner et al., 2017; Piper et al., 2019; Tedersoo et al., 2019).

There are currently very few studies on the holistic interactions between microorganisms and the flax plant during its growth and their implication on the quality of fibers. The only interactions studied for the moment are those taking place during retting, and that concern the capacity of microorganisms to degrade the plant cell wall and therefore *in fine* to affect fiber quality for their future use (Table 3). The first investigation using the metabarcoding approach investigated bacterial communities during kenaf water-retting under different controlled conditions (Visi et al., 2013). The authors used the 16S rRNA gene as the molecular marker and performed sequencing with an Ion Torrent Personal Genome Machine (PGM) system. The operational taxonomic unit (OTU) richness values from Visi et al. (2013) seem to be extremely high compared to other water-retting studies (Table 3). More recently, the evolution of both bacterial and fungal communities during water-retting of kenaf was studied using 18S rRNA gene (Duan et al., 2020). However, the results obtained concerning the Fungi kingdom must be taken with care because precisions concerning the bioinformatics analysis are not available (e.g., sub-sampling size/rarefaction) and certain inaccuracies on the taxonomic affiliation occured (e.g., confusion between the different taxonomic levels; Opisthokonta has been classified as a subkingdom and a phylum). To date, only one study has been conducted on hemp retting by metabarcoding (Law et al., 2020). This study indicated an average OTU richness mean of one hundred. In comparison, the study by Ribeiro et al. (2015) identified 66 different bacterial phylotypes. Despite the fact that these two studies involved different environmental conditions –Law et al. (2020) conducted hemp retting in a greenhouse under controlled conditions, while Ribeiro et al. (2015) collected samples in the field – the OTU or phylotype distributions were similar for the Proteobacteria (85 *vs.* 77%), Bacteroidetes (8 *vs.* 11%) and Firmicute (1% or less) but differed for Actinobacteria (2.3 *vs.* 11%).

Until now, it is flax retting that has been the most studied by metabarcoding, with two studies conducted on field retting (Djemiel et al., 2017) and one on water-retting in tanks (Zhao et al., 2016; Figure 3A). Zhao et al. (2016) focused on the evolution of anaerobic bacteria using a partial 16S rRNA gene sequence region following the addition of a strain of *Bacillus cereus* HDYM-02, previously characterized in their laboratory as being very effective in the decohesion of fibers. Interestingly, this species had been identified previously as an efficient retting agent that did adversely affect fiber mechanical properties (Donaghy et al., 1990). The study by Zhao et al. (2016) demonstrated the power of HTS since they were able to identify five phyla (Actinobacteria, Cyanobacteria, Bacteroidetes, Firmicutes, and Proteobacteria) and eight classes (Flavobacteriia, Sphingobacteriia, Bacilli, Clostridia, Negativicutes, α-proteobacteria, β-proteobacteria, and γ-proteobacteria), some of which were not previously known to be involved in water-retting (e.g., Cyanobacteria and Bacteroidetes for phyla, and Flavobacteriia, Sphingobacteriia, Negativicutes, α-proteobacteria, and β-proteobacteria for classes; Zhao et al., 2016).

The interest of the metabarcoding approach for flax field-retting was also demonstrated by two recent studies (Djemiel et al., 2017; Chabbert et al., 2020). The first study explored the diversity and dynamics of bacterial and fungal communities involved in this process by using an HTS DNA metabarcoding approach (16S rRNA/Internal Transcribed Spacer (ITS) region, Illumina Miseq) on plant and soil samples collected over a period of 7 weeks in July and August 2014 (Djemiel et al., 2017). Twenty-three bacterial and six fungal phyla were identified in soil samples and 11 bacterial and four fungal phyla in plant samples. Dominant phyla identified were Proteobacteria, Bacteroidetes, Actinobacteria, and Firmicutes (bacteria) and Ascomycota, Basidiomycota, and Zygomycota (fungi) all of which have been previously associated with flax dew-retting except for Bacteroidetes and Basidiomycota that were identified for the first time. The use of this powerful method also allowed the identification of rare phyla never previously associated with retting: Acidobacteria, CKC4, Chlorobi, Fibrobacteres, Gemmatimonadetes, Nitrospirae, and TM6 (bacteria), and Chytridiomycota (fungi). These results perfectly illustrate the efficiency of metabarcoding for detecting phyla of low abundance. In addition, this study also revealed that the agricultural practice of swathe turning affected bacterial and fungal community structure, and most likely contributed to a more uniform retting. The use of the prediction tool Phylogenetic Investigation of Communities by Reconstruction of Unobserved States (PICRUSt) (Langille et al., 2013; Douglas et al., 2020); was also used on OTU tables to decipher the functional potential of bacterial communities. This analysis predicted a large collection of potential bacterial enzymes capable of hydrolyzing the backbones and side-chains of cell wall polysaccharides. These results demonstrate the interest of a combined metabarcoding and PICRUSt analysis to predict functional composition of bacterial communities. A similar approach reported by Chabbert et al. (2020) allowed a comparison of the evolution of bacterial and fungal retting communities under different environmental conditions. Eight out of the ten most abundant bacterial OTUs identified in this study were also among the top ten OTUs identified in the 2014 study by Djemiel et al. (2017) in the same geographical area, but in a different field and under different climatic conditions. In contrast, only half of the top ten fungal OTUs were retrieved. In this last study, enzyme activities were measured in addition to targeted metagenomics showing the presence of a high microbial diversity with potential enzymatic functions during retting. The comparison of these two studies could suggest that a year-to-year variability exists, perhaps more so in fungal communities compared to bacteria. Nevertheless, the identification of comparable enzymatic functions in the two studies would suggest a functional redundancy within the communities. In conclusion, the contribution of metabarcoding to the knowledge about the microbiology of retting is undeniable. In addition, the technique can also be adapted to ask questions about the living world in the widest possible way. For example, by refining the choice of primers metabarcoding can also be used to inventory often neglected organisms such as Archaea or microeukaryotes (Bahram et al., 2019).

## Integrative Meta-Omic Approaches to Decipher the Metaphenome and Global Ecology of Dew-Retting

Our understanding of the microbiology of retting has clearly benefited from the use of targeted metagenomics/metabarcoding. In particular, this strategy has made it possible to make a breakthrough in our knowledge about microbial community diversity and structure during this process. However, while metabarcoding allows identification of microorganisms, the functional gene content of the community can only be predicted based on the available knowledge of the whole genome sequences of individual organisms (or phylogenetically related organisms) making up the community. Furthermore, metabarcoding cannot determine which functional genes are expressed. Nevertheless, recent advances in sequencing technologies and computational capabilities offer a unique way for different meta-omic approaches (e.g., metagenomics, metatranscriptomics, and metaproteomics) to fill the gap from taxonomic information to functions by generating information on the functional gene composition of the retting microbial community.

To our knowledge, while there are currently no publications reporting the use of these meta-omics approaches to study retting, recent studies have been performed on biologically similar (but not identical) processes such as litter degradation. This latter phenomenon is indeed commonly described as a breakdown of dead plant material, which is composed of cellulose, hemicellulose, lignin, pectin but also proteins and can therefore be compared to the retting process. In the following sections we present some examples of how different meta-omic approaches have been used to look at plant biomass breakdown and discuss how these approaches could be exploited to provide functional information on retting.

### Metagenomics

Shotgun metagenomics is a high-throughput, culture-independent genomic analysis of all microbes present in a sample. It starts with extraction of total DNA from a sample followed by library preparation and sequencing. Shotgun metagenomics is thus an untargeted global sequencing approach compared to the 16S/ITS/18S rRNA sequencing used for metabarcoding (targeted metagenomics) and which is sometimes erroneously referred to as metagenomics. In the former approach, the aim is to sequence all the genomes present, whereas, in the metabarcoding strategy only the rRNA amplicons are sequenced. Coupled with functional profiling, shotgun metagenomics enables the description of the total genetic content of the microbial community. The functional annotation of the obtained sequences requires the use of several databases, including well-known broad databases such as NCBI nt, ortholog databases such as Clusters of Orthologous Groups (COG) and Evolutionary genealogy of genes: Non-supervised Orthologous Groups (EggNOG) for inference of functional categories, pathway databases such as Kyoto Encyclopedia of Genes and Genomes (KEGG) (Kanehisa et al., 2014) for metabolic pathway reconstruction and specific databases such as CAZy (Lombard et al., 2014) for CAZymes prediction. In a plant biomass context, shotgun metagenomics is principally applied to the mining for novel genes encoding potentially new/interesting CAZymes (Montella et al., 2017) driven by the need to find new biocatalysts for biofuel production. Shotgun metagenomics is also used for whole-genome reconstruction of individual species, the so-called metagenome-assembled genomes (MAGs). This approach allows researchers to simultaneously uncover a microorganism’s functional potential and its identity, with a more resolutive, unbiased phylogenetic information than with metabarcoding. However, the sequencing depth obtained in a sample is critical for successful microbial genome reconstruction and the complexity of the microbial community usually hinders such an approach (Alteio et al., 2020).

With regard to retting, no shotgun metagenomic studies have been conducted to date, and insights into genes involved in fiber degradation are still only obtained via culture-dependent genomic studies (Datta et al., 2020). Nevertheless, other environments associated with cell wall breakdown such as crop soils, and forest soils and litters have been studied (Table 4). Albeit the environmental conditions, cell wall structure and microbial community composition differ from those encountered during fiber retting, such studies show how the genetic potential of cell-wall decomposing microorganisms and their associated functions can be identified using shotgun metagenomics and illustrate how such approaches could be used in future to provide more in-depth information about retting.

**TABLE 4:** Selected metagenomic studies indicating number of reads using different sequencing technologies for a variety of materials from biological situations that present similarities to retting.

**Matrix**	**Methods (Sequencing Technology/Main Bioinformatic tools)**	**Number of Reads (Annotation Information)**	**Taxonomic Groups**	**References**
Soil	454 Pyrosequencing/MG-RAST	1.35 million	Bacteria	Fierer et al. (2012)
Crop soil	454 Pyrosequencing/MG-RAST	∼1,000,000 (53.9% with taxonomic annotation)	Bacteria, Archaea, Fungi	Souza et al. (2013)
Litter	Illumina/MG-RAST	1,265,787 (53% with taxonomic annotation)	Bacteria, Fungi	Berlemont et al. (2014)
Crop soil	454 Pyrosequencing/MG-RAST	∼4 million (54.2% with functional annotation)	Only functional	Souza et al. (2015)
Crop soil	454 Pyrosequencing/Diamond, blastn, hmmscan, MEGAN	∼900,000 (61% with taxonomic annotation, 35.21% with functional annotation)	Bacteria	de Vries et al. (2015)
Litter	Illumina/MG-RAST, blastn	717,933,077 (24-33% with functional annotation)	Bacteria, Fungi	Freedman et al. (2016)
Rainforest and pasture soils	Illumina/MG-RAST, MEGAHIT assembly	6,366,557,730	Bacteria, Archaea	Kroeger et al. (2018)
Crop soil	Illumina/MGX, MEGAHIT, dbCAN2, BlastKOALA, GhostKOALA	505 million	Bacteria, Archaea, Fungi	Nelkner et al. (2019)
Bamboo fiber soaking pit/bamboo pulp pit	Illumina/LBPSDB, BlastP, dbCAN	43 million (∼55% with functional annotation)	Bacteria	Cui et al. (2019)
Rainforest-to-crop soil (mesocosm)	Illumina/MG-RAST	10.7 million	Bacteria	Goss-Souza et al. (2019)
Litter	Illumina/SOAPdenovo, Blastp	∼4 billions	Bacteria, Fungi	Wang et al. (2019)
Forest soil	Illumina/SPAdes, IMG, dbCAN	1,391,343,556	Bacteria, Archaea	Alteio et al. (2020)

In a study of Amazonian rainforest and rainforest-to-pasture converted soils, Kroeger et al. (2018) showed (using a classical shotgun metagenomic strategy) that the microbial community undergoes drastic taxonomic and functional shifts with land-use type. Dominant phyla were common for both soils while less abundant phyla were strongly affected by the land-use type. In the pasture soil, gene functions were predominantly associated with carbohydrate metabolism, dormancy and sporulation, and regulation and cell signaling whereas in the rainforest soil, they were mostly associated with transcription and vitamin production. Although these findings do not constitute a breakthrough on the effect of different land-type use on microbial community composition, the metagenomics analysis was undoubtedly improved by the reconstruction of 28 MAGs from both dominant and rare phyla. Interestingly, a Melainabacteria MAG from a rare lineage was only detected in the pasture soil and shown to contain 72 protein encoding genes not found in other Melainabacteria genomes available. Among the Melainabacteria MAG genes annotated some were potentially involved in carbohydrate utilization. By gaining insights into the Melainabacteria MAG this study provides an example of how shotgun metagenomics can be used to explore the functional diversity of rare biospheres.

A MAG approach can also be applied to explore the functional diversity among members of the same phylum (Alteio et al., 2020). In this study, the authors reconstructed 67 putatively novel Bacteroidetes MAGs from warmed experimental forest soils and showed clade-specific diversity and abundance of CAZymes, suggestive of an extensive potential for polysaccharide degradation among members of the clade. In contrast, 17 unclassified Bacteroidetes MAGs lack major CAZymes required to efficiently deconstruct polysaccharides, suggestive of a rather limited role in polysaccharide degradation.

The study of the effect of agricultural management practices on crop soil microbial communities is also a growing field for the use of shotgun metagenomics and aims to correlate these practices to microbial community taxonomic diversity and the pool of functions (Souza et al., 2018; Nelkner et al., 2019). In their study, Souza et al. (2018) analyzed soil samples that were subjected to four different soil and crop management practices (conventional tillage and no tillage, each with crop succession or crop rotation). Although their results revealed a limited impact of management practice on the abundance of each functional category from the SEED database, the use of KEGG pathways identified some practice-related differences in key enzymes involved in the metabolism of fructose and mannose.

Another interesting example of the use of shotgun metagenomics to understand microbial degradation of plant cell wall polymers concerns the recent work by Kundu et al. (2019). The authors firstly obtained sequences from public shotgun metagenomics datasets of DNA extracted from different gut compartments of the termite *Nasutitermes corniger*. They then combined these data with metabolic data collected in the literature, resulting in a set of 2,988 metabolic reactions, that they used to construct a species-wide lignocellulose network. They identified 15 key bacterial species, all exclusively found in one gut compartment, producing a variety of enzymes that can breakdown lignocellulose including endoglucanases, exoglucanases, endo-1,4-β-xylanase, β-glucosidase, β-xylosidase, and cellobiohydrolase. In turn, the degradation products fed two other key bacterial species involved in fermentation.

### Metatranscriptomics

Shotgun metagenomics offers a unique view of the functional potential (i.e., the genes present) of the microbial community and how it is affected (or not) by its environment. It does not, however, provide any information about the functions that are actually realized (i.e., the genes expressed and translated into functional proteins) by each member of the community. A first step in obtaining this information can be gained from gene expression studies through RNA sequencing. When applied to complex microbial communities this is called metatranscriptomics. Metatranscriptomics provides a snapshot of gene expression and constitutes a first step in going from ‘what the microbial community can do’ to ‘what the microbial community does.’ By analyzing gene expression at the community level, metatranscriptomics allows scientists to determine which metabolic pathways are active as well as their relative importance, thereby enabling predictions to be made about the functional role of the community. In addition, by accumulating metatranscriptomic data over the time course of a biological process it becomes possible to obtain a more accurate picture of the functional dynamics. The principle of metatranscriptomics is similar to the one described above for shotgun metagenomics except that total RNA (and not DNA) from a sample is extracted. The RNA then has to be processed for mRNA enrichment and/or rRNA depletion since rRNA are highly abundant and will compete for sequencing, resulting in poor representation of mRNA. As in all expression studies, particular precautions have to be taken to avoid RNA degradation during sample transport and preparation which can be challenging for field studies. A functional annotation of quality also relies on the use of several databases (including annotated metagenomes) to produce the most comprehensive transcript function prediction possible (Shakya et al., 2019).

As for shotgun metagenomics, metatranscriptomics has not yet been used in the context of retting. Nevertheless, some studies on biologically related environments have been done (Table 5). In their study, Hesse et al. (2015) looked at how CAZymes expression profiles were affected by long-term N deposition from leaf litter in forest soils from two geographically distant sites. They showed that the expression of CAZymes from bacterial or fungal origin responds to N amendment, with both common- and site-specific responses. Nitrogen input tended to favor CAZymes from bacterial origin. The authors also focused on the dynamic balance between Ascomycota and Basidiomycota fungi and showed a decrease of CAZymes from Basidiomycota in N-amended soils compared to ambient soils, that could be possibly linked to an overall decrease in litter decomposition under such conditions. Another valuable information that can be gained from metatranscriptomics concerns the role of other – generally ignored or at best, paid scant attention - members of the microbial community such as protists and viruses. Both of these groups of organisms have the ability to shape the microbial community by interacting with other members, and can thus indirectly modify lignocellulose decomposition activity. Current studies in soils have so far only focused on the taxonomic diversity of protists and viruses, revealing their extreme yet still unexplored diversity (Geisen et al., 2015; Starr et al., 2019). Further studies will be needed to investigate their role and interplay with the whole microbial community.

**TABLE 5:** Selected metatranscriptomic studies indicating number of reads using different sequencing technologies for a variety of materials from biological situations that present similarities to retting.

**Matrix**	**Methods (Sequencing Technology/Main Bioinformatic Tools)**	**Number of Reads (Annotation Information)**	**Taxonomic Groups**	**References**
Forest soil	TA Cloning and sequencing/Blast	119	Eukaryotes	Bailly et al. (2007)
Forest soil	Cloning, Sanger/Blast, Blast2GO, MEGAN	20,000	Eukaryotes	Damon et al. (2012)
Soil and maize leafs (mesocosms)	454 Pyrosequencing/Blast, HMMSearch, MEGAN	171,184 (∼30% annotated)	Fungi	Kuramae et al. (2013)
Mineral, litter and peat soils	454 Pyrosequencing/Blast, MEGAN	32,808 (only taxonomic analysis)	Protists	Geisen et al. (2015)
Forest soil	Illumina/Blastx, MEGAN	∼200 million	Bacteria, fungi	Hesse et al. (2015)
Higher termite gut	Illumina/CLC genomics workbench, dbCAN, IMG-MER	112 million	Prokaryotes	Marynowska et al. (2017)
Termite gut	Illumina/Trinity, Blast	14.3 million (44.5% of 71,117 unigenes)	Eukaryotes	Geng et al. (2018)
Soil amended with ground wild oat (microcosm)	Illumina/HMMER, MAFFT, USEARCH	(3,884 viral sequences)	Viruses	Starr et al. (2019)

Thanks to advances in sequencing technologies and analysis tools in the last decade, more and more shotgun metagenomics and metatranscriptomics data can be obtained and analyzed thereby enabling in depth descriptions of microbial community structure and functions. The complexity of some environments such as soil that was previously a major drawback for analysis can now be alleviated by using other techniques to experimentally reduce the complexity of the community (Alteio et al., 2020). Despite current challenges, these approaches can be viewed, more than ever, as essential strategies on the path to decipher the role of microbial communities, and constitute a basis for other non-genomic-based omics approaches.

### Metaproteomics

Like metabarcoding and metagenomics, metaproteomics can be used to provide information about microbial community structure and the different factors that can modify it. For example, it was recently shown that the long-term application of fertilization led to significant changes in microbial community structure and function associated with increased microbial biomass (Wu et al., 2015). In another example, the use of a stable isotope-probing metaproteomics approach using ^15^N-labeled plant-derived organic matter in a soil microbial community provided novel information on community structure and microorganism feeding strategies (Starke et al., 2016). This study revealed that Proteobacteria *w*as the most abundant phylum followed by Actinobacteria and Ascomycota. It also demonstrated copiotrophic behavior for Rhizobiales belonging to Proteobacteria, Actinomycetales belonging to Actinobacteria and Chroococcales belonging to Cyanobacteria as these phylotypes immediately incorporated ^15^N from the added plant tissue. Conversely, the fungal Saccharomycetales and the bacterial Enterobacteriales, Pseudomonadales, Sphingomonadales, and Xanthomonadales displayed slower ^15^N-assimilation. Such a strategy could also be expected to generate information about the retting process in flax and other fiber species.

While metatranscriptomics provides information about the genes expressed in a community only a metaproteomics approach will provide precise information about the different proteins responsible for the observed function (in this case cell wall degrading enzymatic activity) in a given habitat (Herbst et al., 2016). Recent biochemical studies (Bleuze et al., 2018; Chabbert et al., 2020) have highlighted the role of different enzyme families during hemp and flax retting, but they did not identify the proteins responsible for these activities. The astounding advances of the past decade that have led to fast and affordable protein sequencing technologies associated with the increase in database information have led to a rapid increase in the number of protein identifications (Table 6). Protein data will not only give more direct information about microbial activities compared to metagenomics and metatranscriptomics, but can also fill taxonomical gaps of nucleotide-based methods (Wilmes and Bond, 2004, 2006).

**TABLE 6:** Selected studies performed on biologically-similar to retting systems reporting detection or identification of proteins using different proteo- and metaproteomic methods.

**Matrix**	**Proteome Analysis Methods**	**Number of Proteins**	**References**
Greenhouse soils	1DEa N-terminal sequencing GeLC-MS/MS	5	Murase et al. (2003)
Compost soil	GeLC-MS/MS	4	Benndorf et al. (2007)
River gravel or lava granules	2DE	240	Benndorf et al. (2009)
Agricultural soil	2DE	∼250	Chen et al. (2009)
± Toluene-amended soil microbial ± inoculated cultures	1D MALDI-TOF/TOF MS	47	Williams et al. (2010)
Grassland soil	2D-LC-MS/MS	333	Chourey et al. (2010)
Beech leaf litter	GeLC-MS/MS	Up to 1,724	Schneider et al. (2010, 2012)
Soil microcosms spiked with *Cupriavidus metallidurans* proteins	2DE	320	Giagnoni et al. (2011)
Rice rhizosphere soil	2DE MALDI-TOF/TOF-MS	122	Wang et al. (2011)
*Rehmannia glutinosa* rhizosphere Soil	2DE MALDI-TOF/TOF-MS	103	Wu et al. (2011)
Agricultural abandoned soils	GeLC-MS/MS	11–71	Bastida et al. (2012)
Forest soil (FS) and potting soil (PS)	2D-LC-MS/MS	(FS) 226–494 (PS) 80–237	Keiblinger et al. (2012)
Batch fermentation media by *Ruminiclostridium cellulolyticum*	LC-MS/MS	1,194	Badalato et al. (2017)
Paddy soil	2DE MALDI-TOF MS	∼300	Wu et al. (2015)
Soil mesocosm	1D UPLC-LTQ Orbitrap Velos MS/MS	Diversity information	Starke et al. (2016)
Soils under semi-arid climate	Orbitrap MS	Diversity information	Bastida et al. (2018)
Silty-loam soil	2D LC-MS/MS	Up to ∼4,000	Callister et al. (2018)
Decayed pine	LC-MS/MS	1,964	Hori et al. (2018)
Kenaf bast	iTRAQ labeling 2D-LC-MS/MS	197	Duan et al. (2020)
Decayed beech dead wood	LC-MS/MS	Up to ∼1600	Kipping et al. (2020)

As is the case for metagenomics and metatranscriptomics, there are actually very few descriptions of a metaproteomics approach being applied to flax retting. Nevertheless, some general conclusions and useful information can be obtained from the studies on biologically similar systems. In a recent metaproteomics study of lodgepole pine decay, a diverse array of carbohydrate-active enzymes (CAZymes) were identified, representing a total of 132 families or subfamilies among which 672 glycoside hydrolases (GHs) including highly expressed cellulases or hemicellulases (Hori et al., 2018). The authors suggested that the observed enzymatic diversity and the coexistence of brown and white rot fungi indicated the existence of complex interactions between fungal species and degradation strategies. Another very recent metaproteomics study on the degumming of kenaf bast (outer-stem tissue containing fibers) probably represents the biological situation most closely related to retting (Duan et al., 2020). Here the authors analyzed kenaf bast fragments immersed into microbial fermentation liquid collected from different sites. Microbial secretomics analysis identified 197 proteins, including 67 differentially expressed proteins (DEPs) including Rds1, pyruvate kinase I and aconitate hydratase peptides. However, no DEPs associated with the degradation of cell wall polymers were observed underlying the difficulty of identifying non-core metabolism proteins.

Another important biological interest of using metaproteomics to study retting concerns the identification of protein termini for maturation or proteolytic processing (Westermann et al., 2017). After translation, proteins undergo a maturation step to obtain their final, active conformation. Apart from chemical modification of amino acid side chains, this maturation often involves cleavage of the polypeptide resulting in new N- or C-termini. The best-known example of this is the removal of the signal peptide from the N-terminus of the majority of secreted proteins, including most proteins involved in lignocellulose degradation. Of interest is the fact that non-signal peptide proteolytic processing has been described for plant cell wall interacting enzymes from the fungal genera *Cladosporium* and *Cryptococcus* (Ritch et al., 1991; Amoresano et al., 2000), identified as dominant during hemp retting (Ribeiro et al., 2015). Studies on proteins from *Trichoderma reesei* (Ståhlberg et al., 1988) the best studied cellulolytic fungi, indicate that proteolytic events are important in the control of the activity of cell wall lytic proteins (Coller et al., 1998). Similar observations have been made for bacterial enzymes with potential involvement in the partial degradation of plant cell walls, for instance, Erwinia pectate lyases are activated by N-terminal processing (Shevchik et al., 1998), as are Pseudomonas endoglucanases (Huang et al., 1989). For the study of proteolytic processing, different targeted approaches have been developed, the flagship being COFRADIC: Combined Fractional Diagonal Chromatography (Marino et al., 2015; Tanco et al., 2015) although more accessible alternatives have also been developed (Schilling et al., 2010).

### Meta-Omics – Limitations and Best Practices

Before undertaking any experimental steps of any meta-omics approaches, it is firstly necessary to clearly define the biological question and research objectives (Matallana-Surget et al., 2018) in order to determine which meta-omics approach is best adapted.

While metabarcoding is an extremely interesting tool for identifying bacterial, fungal or other communities within a given sample, one of the main limitations of this approach is that it only delivers relative population data and is not quantitative (Lamb et al., 2019) regardless of the methodology, the primers, or the sequencing platform used. Inclusion of mock communities during metabarcoding is one way of getting around this problem. Metabarcoding is based on PCR amplification that can generate chimeras or other bias linked to this method and denoising data is therefore an important step in analysis that should be done with care. Recent pipelines (Qiime2 AND DADA2; Hall and Beiko, 2018) based on the notion of ASV (Amplicon Sequence Variant) are less likely to induce false discovery and seem more appropriate to identify diversity, replacing the notion of OTUs built by clustering. Generally, the errors and uncertainties associated with metabarcoding can often be tempered by careful study design, appropriate primer choice and robust sampling and replication (Murray et al., 2015). For example, the mix of plant and microorganism material collected during retting requires a careful choice of primer design to prevent the amplification of chloroplast/mitochondrial DNA (Djemiel et al., 2017). Another limitation of metabarcoding is its inability to describe the functional gene content of the community despite advanced functional inference tools such as PICRUSt2 (Douglas et al., 2020). Such information can only be predicted from whole genome sequence data of individual phylogenetically related organisms. However, when considering the cost/quantity of information ratio, metabarcoding is undoubtedly the most affordable way to approach the microbiology of retting.

In contrast to metabarcoding, shotgun metagenomics provides information about the functional potential (i.e., the genes present) in the microbial community. This approach is of course much more informative than metabarcoding but requires a much greater sequencing depth and therefore a much higher cost. Likewise, the need to assemble large-scale sequencing data requires skills and adequate computer resources. Extensive reviews of best-practices can be found in the literature and provide useful guidelines, from study design to data analysis, and address common pitfalls of such an approach (Knight et al., 2018; Bharti and Grimm, 2019).

If shotgun metagenomics can answer the questions “who is there” and “what enzymatic potential” is present, it cannot, however, provide any information about the functions that are really present (i.e., the genes expressed and translated into functional proteins). Such information can be obtained by metatranscriptomics. Despite the fact that the concept of metatranscriptomics has been around for several years (Gorni, 2013), there is currently no established protocol for this technique. Nevertheless, a number of recommendations can be made of which the most important is probably the necessity for ribodepletion in order to remove rRNAs from samples subjected to sequencing. This step remains critical and must be carefully controlled as the approaches used are not always successful (Barua, 2017). As with shotgun metagenomics, computer processing - in particular the assembly of the metatranscriptome, as well as the cost associated with sequencing remains an obstacle to the deployment of this approach on a large scale. Best practices for metatranscriptomics, especially regarding data analysis, are now starting to be published (Shakya et al., 2019), as the evolution of analysis tools grows.

If metatranscriptomics provides data on gene expression in microbial communities only metaproteomics can provide information about the different proteins responsible for the observed biological activity of the community (Herbst et al., 2016). Whatever the origin of the microbiome (human, plant, soil…), successful shotgun metaproteomics still faces a number of conceptual and technological hurdles that need to be overcome (Blackburn and Martens, 2016; Heyer et al., 2017; Knight et al., 2018; Matallana-Surget et al., 2018; Abiraami et al., 2020). Currently, a major limitation for metaproteomics in such systems is the lack of effective and reproducible protein extraction protocols and standardized data analyses resulting from i) the tremendous heterogeneity of samples (i.e., plant, soil and litter), ii) the low protein yield that can be obtained from soil and litter matrices and iii) the wide range of protein abundance levels, Becher et al. (2013), Keiblinger et al. (2016), Keiblinger and Riedel (2018).

Another issue concerns the size and the complexity of multi-organism protein sequence databases that are likely to contain many highly similar orthologs (Blackburn and Martens, 2016). There is therefore a global need for the improvement of database quality, including grouping of redundant proteins as well as taxonomic and functional annotation. A possible solution might be the generation of non-redundant fusion metagenomes for each type of microbial community (May et al., 2016). The use itself of protein databases could also be standardized, since some researchers use comprehensive protein databases and others use diverse metagenomes, which differ in the processing state and origin (Heyer et al., 2017). Currently, state-of-the- art metaproteomics studies only achieve identification of 5–30% spectra (Heyer et al., 2017) and it can be expected that a closer cooperation between bioinformaticians and biologists will improve bioinformatic strategies and increase the number of identified spectra. In the future, metaproteome studies will not only enable researchers to precisely characterize members of microbial and fungal communities but will also allow them to identify the retting hydrolytic enzymatic potential under given conditions. Approaches linking phylogenetics and functionality, could also be expected to help gain deeper insights into terrestrial microbial ecology.

In conclusion, by using integrative meta-omic approaches such as meta-genomics, metatranscriptomics and metaproteomics, a much better characterization and understanding of the dynamics of the retting microbial community and its molecular interactions can be obtained. However, for all methods, the same ‘bottlenecks’ remain: standardization of appropriate experimental designs to deal with the tremendous heterogeneity of samples, the use of state-of-the-art methodologies coupled with optimal genomic sequence information, pertinent database selection, and appropriate bioinformatic tools to analyze, integrate and visualize comprehensive global data sets (Becher et al., 2013; Tanca et al., 2013; Allen White et al., 2017). As indicated above, each technique focuses on a subset of the biological interaction network, therefore combining such contemporary molecular tools with eco-physiological studies in a trait-based framework (Krause et al., 2014) will allow scientists to more precisely decipher the global ecology of dew-retting. In a similar approach to that used for soil or rhizosphere microbial communities (El Amrani et al., 2015; Rochfort et al., 2015) the further inclusion of meta-metabolomic techniques would ultimately allow access to the signaling network thereby leading to an even deeper characterization and associated prediction of the retting metaphenome. Nevertheless, the interpretation of such large sets of data is notoriously difficult, mainly because of the difficulty of successfully integrating huge amounts of data. This difficulty underlines the absolute necessity to develop new methods for investigating dynamic aspects of large-scale models (Zivy et al., 2015; do Amaral and Souza, 2017; Wang et al., 2018) in a more integrative way (Lê Cao et al., 2008; Rohart et al., 2017).

## Concluding Remarks and Future Perspectives

A multitude of intrinsic and extrinsic factors, both during plant growth and after harvest, all contribute to establishing the final phenotype of plant fibers reaching the factory (Figure 1). Since these factors affect fiber morphology, cell wall composition and organization they will also impact on the behavior of the separated fibers during subsequent industrial processing. In this paper we have focused on the different meta-omic technologies that have been, or could be, exploited to improve our understanding of one of these many factors - the field retting step of flax. This step involves a complex interaction between the harvested plant material and microorganisms, both in the soil and on the plant at the time of harvest. However, the complexity of this interaction does not stop there since the nature of the plant material itself (e.g., fiber morphology and cell wall composition), as well as the composition and functional capacity of the microbiome are also impacted by genetics and environmental conditions. In the light of such observations it is becoming clear that not only do we need to implement a multi-omics systems biology approach, but that we also need to take into account all of the actors that contribute to producing the final phenotype. This idea is embodied in the concept of the ‘holobiont’ which states that we should no longer consider the plant as an isolated ‘stand-alone’ organism, but rather as an individual with its associated microbial communities and in which their overall interactions are modulated by the pedo-climatic environment (Simon et al., 2019). Their association plays a decisive role in terms of the biodiversity and functionality of this ecosystem in which all the partners influence each other. Moreover, plant/microorganism (symbiotic, pathogenic, saprophytic) and microorganism/microorganism interactions will be affected by different biotic factors (e.g., species, cultivar, age, health, and stages of plant development) and abiotic factors (e.g., soil physicochemical composition, climatic conditions (Comeau et al., 2020; Fitzpatrick et al., 2020). As demonstrated in this review the combination of related ‘-omics’ such as metatranscriptomics and metaproteomics, together with biochemical studies, offers the unique opportunity to investigate the particular holobiont constituted by the overall fiber plant growing cycle. There is no doubt that the comprehension of this complex ecological machinery promises to make a major contribution to the control of plant natural fiber quality. For this purpose, it will be necessary to integrate together large multiscale data sets consisting of fiber parameters, biological and environmental information with the development of predictive models to be processed by AI analyses. The collection of massive data would rely on the development of connected microsensors reporting different parameters in real time directly from the field. Flax farmers will then have at their disposal a ‘retting toolbox’ making use of molecular markers, metabolite, protein and gene expression, biochemistry and phenotyping (morphological and agronomic) data for targeting selected traits. The cultivation of flax will then enter the era of smart connected agriculture.

## Author Contributions

SG and SH revised the manuscript. All authors were involved in the conceptualization of the manuscript and wrote the manuscript. All authors approved the final version of the manuscript.

## Conflict of Interest

The authors declare that the research was conducted in the absence of any commercial or financial relationships that could be construed as a potential conflict of interest.

## Footnotes

1 https://phytozome-next.jgi.doe.gov
